# Physical activity trajectories after mild ischemic stroke and their association with 6-month functional outcomes

**DOI:** 10.3389/fneur.2026.1919846

**Published:** 2026-10-22

**Authors:** Wei Zheng, Yixian Zhang, Huayao Huang, Houwei Du, Nan Liu

**Affiliations:** 1 Stroke Research Center, Department of Neurology, Fujian Medical University Union Hospital, Fuzhou, Fujian, China; 2 Department of Rehabilitation, Fujian Medical University Union Hospital, Fuzhou, Fujian, China; 3 Institute of Clinical Neurology, Fujian Medical University, Fuzhou, Fujian, China; 4 Department of Neurology, Fujian Provincial Geriatric Hospital, Fuzhou, Fujian, China

**Keywords:** functional outcome, mild ischemic stroke, modified rankin scale, physical activity, trajectory

## Abstract

**Background:**

Whether post-stroke physical activity (PA) patterns are associated with stroke prognosis remains unclear. We aimed to examine the association between post-stroke PA trajectories and 6-month functional outcomes in patients with mild ischemic stroke.

**Methods:**

This prospective cohort study included patients with mild ischemic stroke (National Institutes of Health Stroke Scale [NIHSS] score ≤ 5) admitted to a teaching hospital between July 2024 and March 2025. PA was assessed using the Saltin-Grimby Physical Activity Level Scale at pre-stroke, 1, and 3 months. PA trajectories from pre-stroke to 3 months post-stroke were identified using growth mixture modeling, yielding three PA trajectories: stable low, increased, and decreased. The primary outcome was functional independence (defined as a score of 0–2 on the modified Rankin Scale; range, 0–6, with higher scores indicating more severe disability) at six months. Multivariable logistic regression was used to examine the associations between PA trajectories and the primary outcome, adjusted for prespecified baseline covariates (age, sex, hypertension, diabetes, and atrial fibrillation).

**Results:**

Among 502 patients (median age, 66 years [IQR 57–72]; 354 males [70.52%]), functional independence at 6 months was achieved in 166 patients with stable low PA (80.58%), 177 with increased PA (88.06%), and 74 with decreased PA (77.89%). The overall three-level test for the trajectory variable was not statistically significant (*P* = 0.086). In an exploratory pairwise comparison, compared with decreased PA, the odds of functional independence were 2.03 (95% CI 1.05–3.90; *P* = 0.034) for increased PA and 1.29 (95% CI 0.70–2.36; *P* = 0.418) for stable low PA.

**Conclusions:**

Although the overall trajectory test was not significant, an exploratory pairwise comparison suggested that an increased PA trajectory was associated with better functional independence compared with a decreased trajectory. These findings should be considered exploratory and require external validation; causality cannot be inferred.

## Introduction

Mild ischemic stroke accounts for approximately one-third of acute ischemic cerebrovascular events (1). Although patients with mild ischemic stroke generally have mild initial neurological deficits, stroke recurrence and long-term functional impairment remain threats to their health and quality of life (2). Evidence from the Platelet-Oriented Inhibition in New TIA and Mild Ischemic Stroke (POINT) trial (3) suggests that mild ischemic stroke should not be regarded as uniformly benign, as a substantial proportion of patients continue to experience disability during follow-up.

Physical activity (PA) has been consistently associated with better functional recovery among stroke survivors. Population-based evidence showed that changes in PA over time were associated with a higher risk of incident ischemic stroke, highlighting the clinical importance of PA as a dynamic exposure (4). Higher levels of pre-stroke PA are associated with either milder stroke severity or smaller infarct volume at stroke onset, and fewer post-stroke complications, lower mortality, and favorable long-term outcomes (5–7). Post-stroke PA may facilitate neurological recovery through multiple mechanisms, including improved cerebral blood flow, vascular risk factor control, and enhanced neuroplasticity (8, 9).

Several recent studies have examined post-stroke PA trajectories using repeated measurements and demonstrated their prognostic relevance (10–12). However, most previous research (13, 14) relied on single measurements that may not capture the dynamic nature of recovery after mild stroke (15). To our knowledge, no study has specifically examined PA trajectories in first-ever mild ischemic stroke with a retrospectively assessed pre-stroke PA measurement and a shorter-term trajectory window (pre-stroke to 3 months) predicting 6-month functional outcomes. Clarifying such associations may help optimize individualized management and inform rehabilitation strategies.

To our knowledge, whether pre-stroke to 3-month PA trajectories relate to functional outcomes in patients with stroke remains unclear. We aimed to identify PA trajectories from pre-stroke to 3 months post-stroke and examine their association with 6-month functional outcomes. We hypothesized that patients with an increased PA trajectory would have a higher likelihood of achieving a favorable functional outcome.

## Study population

This prospective cohort study enrolled adult patients (aged 18–80 years) with first-ever acute mild ischemic stroke admitted to Fujian Medical University Union Hospital between July 2024 and March 2025. Mild stroke was defined as a National Institutes of Health Stroke Scale (NIHSS) of 0–5 (16). To be eligible, participants must have a diagnosis of acute ischemic stroke confirmed by computed tomography (CT) or magnetic resonance imaging (MRI), and be admitted within 72 h of symptom onset. Exclusion criteria included: (1) refusal to participate in physical activity assessments; (2) severe cardiopulmonary disease or other conditions limiting PA; and (3) severe cognitive impairment or psychiatric disorders precluding follow-up (Figure 1).

**Figure 1:**
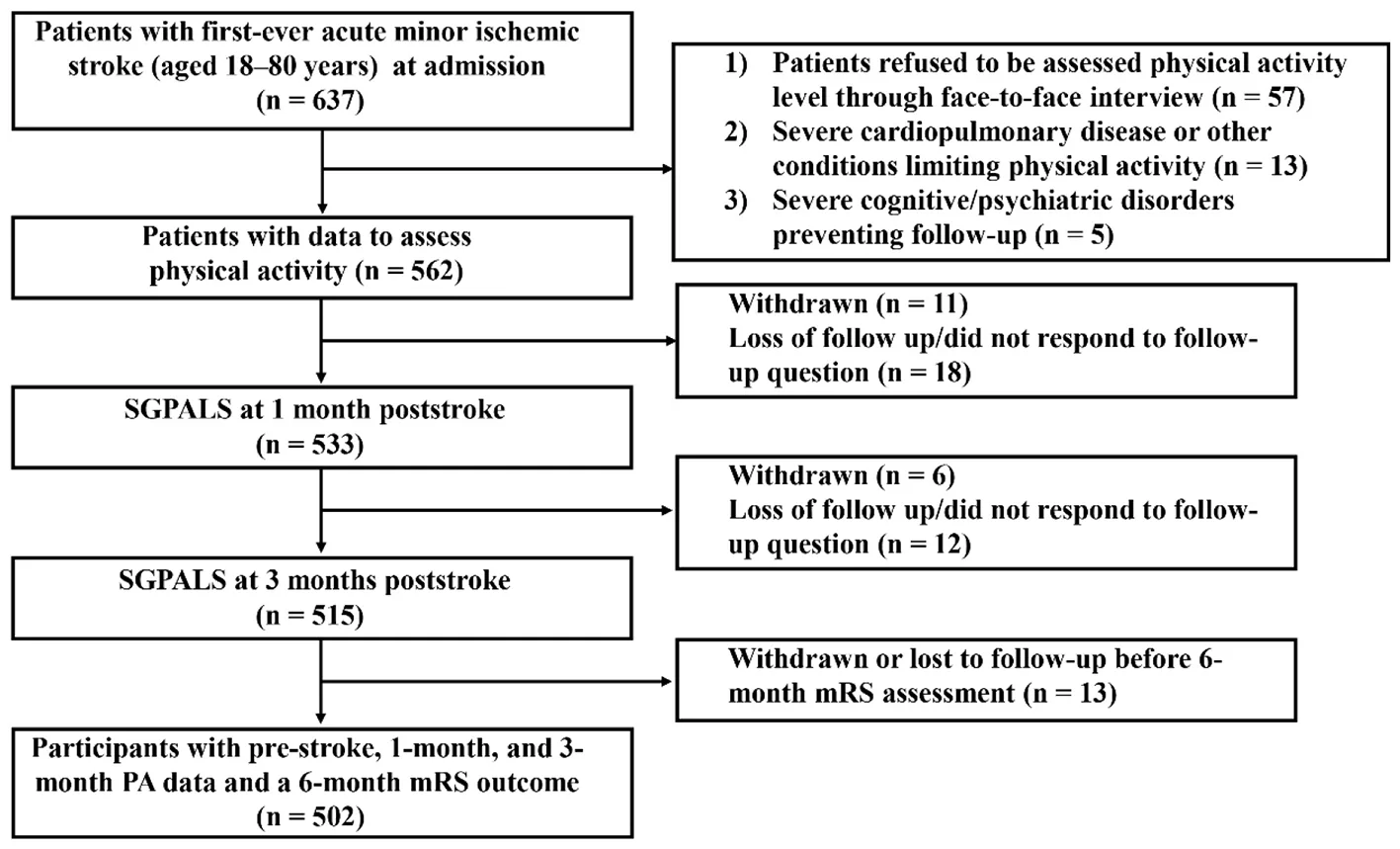
Flow chart.

The study protocol was approved by the Ethics Committee of Fujian Medical University Union Hospital (No. 2023KY030). Written informed consent was obtained from all participants or their legal representatives. We conducted this study in accordance with the Declaration of Helsinki.

## Physical activity assessment

PA was assessed using the Saltin-Grimby Physical Activity Level Scale (SGPALS) (17), a validated self-reported four-level measure of habitual leisure-time physical activity. The four levels were defined as follows: level I, physically inactive; level II, light physical activity for at least 4 h per week; level III, regular physical activity and exercise for at least 2–3 h per week; and level IV, vigorous exercise or competitive sports performed several times per week. For trajectory modeling, PA was assessed at three time points: T0 (at admission, assessing 1 week before stroke onset, retrospectively reported), T1 (1 month after stroke), and T2 (3 months after stroke). At T1 and T2, patients were asked to recall their physical activity during the past month. All follow-up assessments were conducted either face-to-face during scheduled outpatient visits or by telephone when in-person visits were not feasible; no proxy assessments were used.

## Outcome assessment

Functional outcome was assessed at 6 months after stroke onset using the modified Rankin Scale (mRS), a 7-point scale ranging from 0 (no symptoms) to 6 (death), with lower scores indicating better functional status (18). All mRS evaluations were performed by trained neurologists who were blinded to the patients' PA responses and trajectory classification. The primary outcome was functional independence, defined as a mRS score of 0–2 at 6-month (19). The secondary outcome was an excellent outcome, defined as a mRS score of 0–1.

## Data extraction and covariates

Two investigators (W.Z. and Y.Z.) independently extracted baseline data from the prospectively maintained stroke registry and electronic medical records using a standardized data collection sheet. Covariates were selected a priori using a directed acyclic graph (DAG) informed by clinical knowledge and previous literature to estimate the association between physical activity trajectory and 6-month functional outcome (20). The DAG included demographic and socioeconomic factors, pre-stroke health behaviors, pre-stroke health status, stroke characteristics, vascular comorbidities, acute treatment and baseline stroke severity. Specifically, demographic included age and sex; socioeconomic factors included residence and insurance type; pre-stroke health behaviors included smoking status and alcohol status; pre-stroke health status included pre-stroke mRS (retrospectively assessed); vascular comorbidities included diabetes, atrial fibrillation, hypertension, ischemic heart disease and chronic heart failure; stroke characteristics included Trial of Org 10172 in Acute Stroke Treatment (TOAST) subtype (21); acute treatment strategies included intravenous thrombolysis; and baseline stroke severity included the initial NIHSS score and wake-up stroke.

Smoking status was classified as never, current, or former based on smoking behavior during the previous 6 months. Alcohol status was categorized as regular drinking (>once per week), occasional drinking, or never drinking. Hypertension was defined as systolic blood pressure ≥140 mmHg, diastolic blood pressure ≥90 mmHg, self-reported physician diagnosis, or current antihypertensive treatment (22). Diabetes was defined as a history of diabetes, use of glucose-lowering medications, fasting blood glucose ≥7.0 mmol/L, or non-fasting blood glucose ≥11.1 mmol/L (23). Atrial fibrillation was defined by a documented history or electrocardiographic evidence during hospitalization (24). Ischemic heart disease was identified by a documented history of myocardial infarction, angina, or coronary artery disease. Chronic heart failure was defined as a prior clinical diagnosis recorded in the medical history or confirmed by a cardiologist (25). Stroke subtype was classified using the TOAST criteria (21).

## Statistical analysis

PA trajectories from pre-stroke to 3 months post-stroke were identified using linear growth mixture modeling (GMM) (26), as only three time points (T0, T1, T2) were available and a quadratic model was not identifiable. The optimal number of latent trajectory groups was determined based on model fit and clinical interpretability, including the Bayesian information criterion (BIC), absence of very small classes, and clinical meaningfulness of the identified patterns (27). Missing data were handled using multiple imputation with chained equations (MICE) (28). Detailed descriptions of the imputation procedure, including variables with missing values, missingness proportions, number of imputations, imputation model, and pooling methods, are provided in Supplementary Table S1. Continuous variables were presented as mean with standard deviation if normally distributed and median with interquartile range (IQR) if not normally distributed. Categorical variables are summarized as frequencies with percentage. Between-group differences were assessed using the t-test or Kruskal-Wallis test for continuous variables and the chi-square test or Fisher exact test for categorical variables, as appropriate. Associations betw‘een PA trajectories and 6-month functional outcomes were examined using multivariable binary logistic regression models, with the decreased PA group as the reference category. Based on the DAG (Figure 2; DAGitty code provided in the Supplementary materials), the primary covariate-adjusted model (Model 2) included the following baseline confounders: age, sex, hypertension, diabetes, and atrial fibrillation. Admission mRS, NIHSS, thrombolysis, and wake-up stroke are all post-stroke variables and were therefore not included in the primary model; they were included together in a sensitivity analysis. Results are reported as odds ratios (ORs) with 95% confidence intervals (CIs).

**Figure 2:**
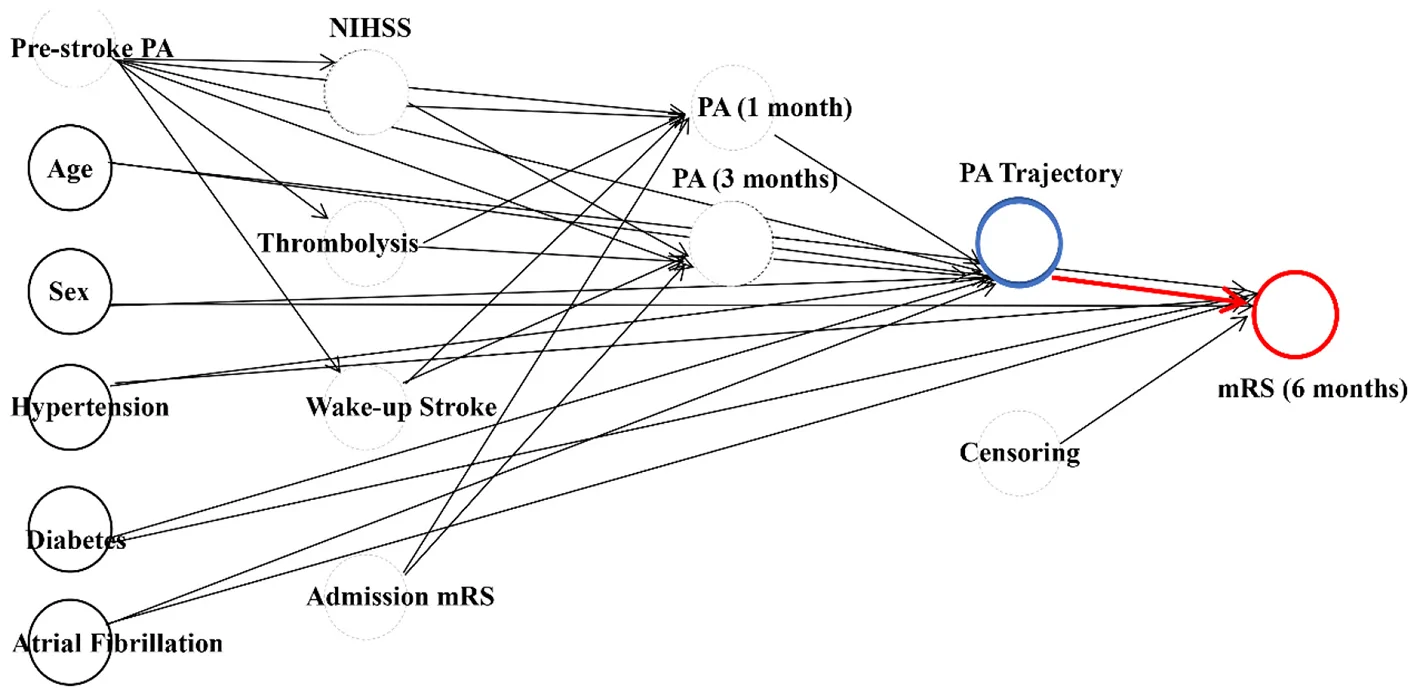
Directed acyclic graph (DAG) illustrating the assumed relationships among physical activity trajectories, functional outcomes, and covariates.

Subgroup analyses stratified by age and sex were *post-hoc*/exploratory and not pre-specified. The age cut-off of 60 years was chosen based on the median age of the cohort. Covariates included in each subgroup model were identical to the primary Model 2, excluding the stratifying variable. Interaction terms were fitted by including the product term (trajectory × subgroup variable) in the logistic regression model, and the Wald test was used to assess significance. Four sensitivity analyses were performed to assess the robustness of the main findings. First, we additionally adjusted for acute-phase variables—NIHSS, intravenous thrombolysis, admission mRS, and wake-up stroke—to assess whether the association was sensitive to over-adjustment for potential mediators. Second, E-values were calculated to assess the potential impact of unmeasured confounding (29). Third, SGPALS scores at 3 months were treated as ordered categorical variables (levels 1–4) using ordinal logistic regression, with level 1 as the reference, adjusted for the same covariates as Model 2. Fourth, to address classification uncertainty, we performed a posterior-probability-weighted logistic regression as a sensitivity analysis. Each participant contributed three records corresponding to the three latent trajectory classes. The weight for each record was the posterior probability of belonging to that class, and the three weights summed to 1 for each participant. The reference class was Decreased. Because records from the same participant were correlated, standard errors were calculated using survey-design robust estimators with participant ID as the clustering variable. This analysis was adjusted for age, sex, hypertension, diabetes, and atrial fibrillation. This analysis partially accounts for classification uncertainty but is not a formal correction for latent class misclassification. All tests were 2-sided, and *P* < 0.05 was considered statistically significant.

## Results

Among 637 patients, we excluded 57 patients who declined to participate in the face-to-face physical activity interview, 13 with severe cardiopulmonary disease or other disabling comorbidities, and 5 with severe cognitive impairment or psychiatric disorders that precluded follow-up. A total of 562 patients completed the baseline PA assessment. After excluding 60 patients who withdrew or were lost to follow-up, 502 patients who had pre-stroke, 1-month, and 3-month PA data and a 6-month mRS outcome were included in the final analysis. The study flow chart is shown in Figure 1. Among the 60 excluded participants, no deaths were documented based on medical record review and death registry linkage. However, detailed baseline characteristics and 6-month functional outcomes were not available for these participants due to loss to follow-up or withdrawal (Supplementary Table S2).

## Physical activity trajectories

GMM identified three exploratory PA trajectory classes from pre-stroke to 3 months post-stroke as the optimal solution, based on model fit indices, class size, and clinical interpretability (Supplementary Table S3). This three-trajectory model had the lowest BIC, sample-size adjusted Bayesian information criterion (SABIC), and Akaike information criterion (AIC) values among the clinically meaningful models and showed moderate entropy (26, 27)_**.**_

A total 206 (41.0%) patients maintained consistently low PA across all time points (stable low PA). A progressive decline in PA (decreased PA) was reported in 95 (18.9%) patients. A total of 201 (40.0%) patients showed a steady increase in PA (increased PA). The estimated trajectory patterns are shown in Figure 3. The distribution of 6-month modified Rankin Scale scores across the three physical activity trajectories is shown in Figure 4.

**Figure 3:**
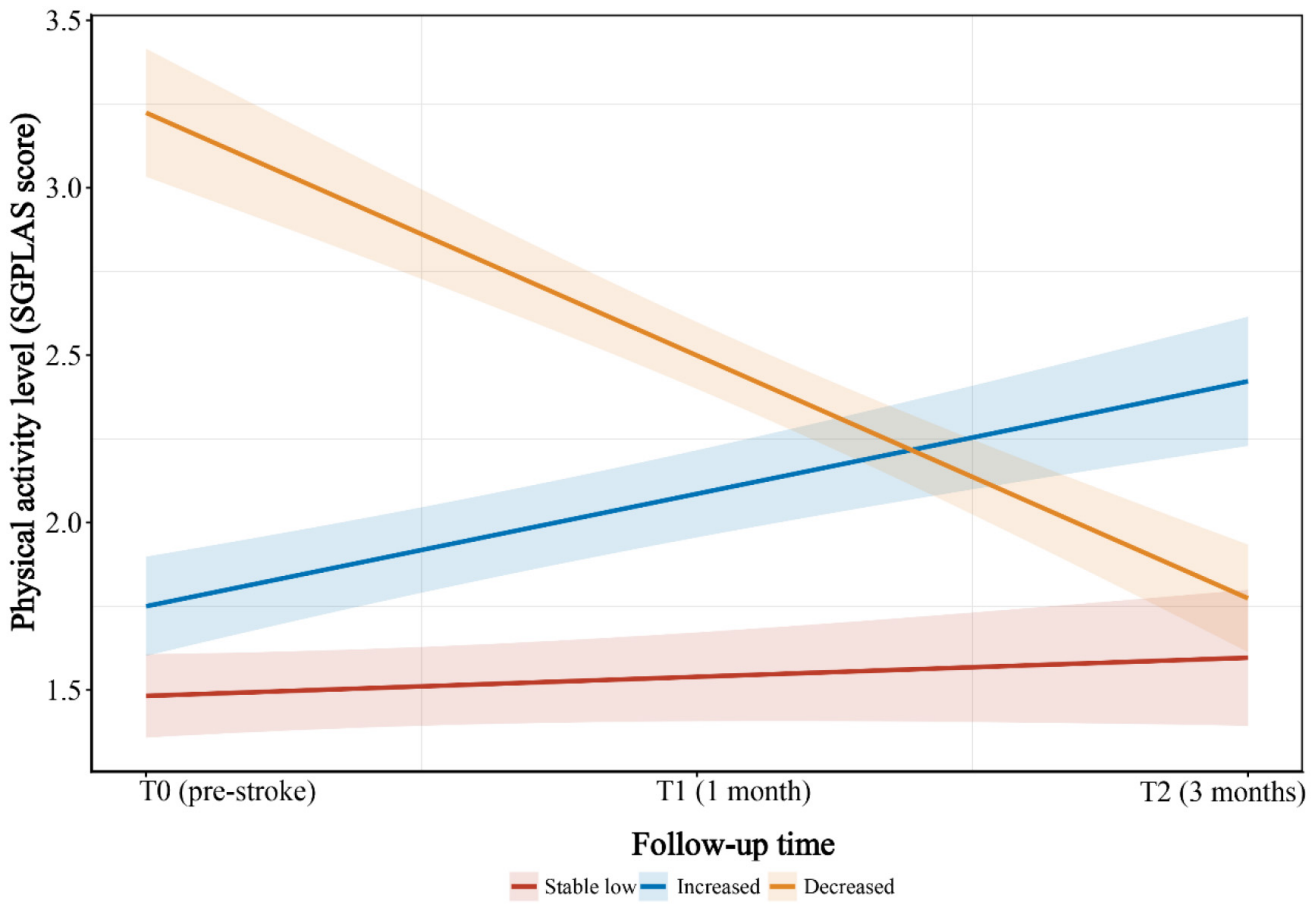
Estimated trajectories of physical activity (SGPALS) from pre-stroke to 3 months post-stroke.

**Figure 4:**
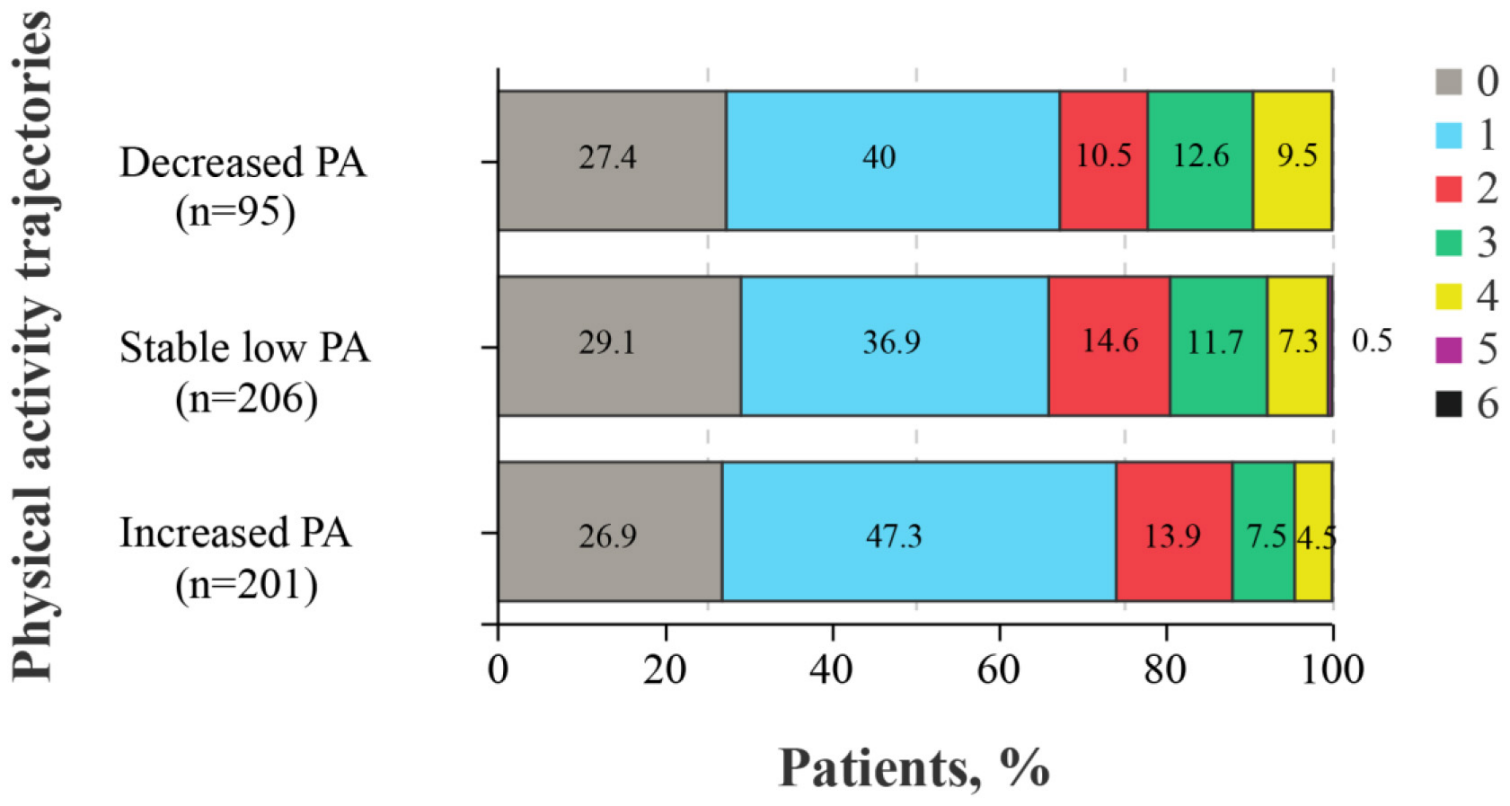
Distribution of 6-month modified rankin scale scores by physical activity trajectory.

## Baseline characteristics by trajectory group

Baseline characteristics stratified by PA trajectories are shown in Table 1. Functional independence at 6-month was observed in 166 patients (80.58 %) with stable low PA, 74 patients (77.89 %) with decreased PA, and 177 patients (88.06 %) with increased PA.

**Table 1:** Baseline characteristics and 6-month outcomes of patients according to physical activity trajectory groups.

Variables	Total (*n* = 502)	Stable low (*n* = 206)	Decreased (*n* =95)	Increased (*n* = 201)
Age, y (median, IQR)	66(57,72)	69 (61,74)	65 (58,72)	62 (55,69)
Sex, n (%)				
Female	148(29.48)	67 (32.52)	26 (27.37)	55 (27.36)
Male	354(70.52)	139 (67.48)	69 (72.63)	146 (72.64)
Insurance type, n (%)				
Urban employee insurance	264(52.59)	116 (56.31)	38 (40.00)	110 (54.73)
Non-urban employee insurance	231(46.02)	88 (42.72)	54 (56.84)	89 (44.28)
Self-paid	7 (1.39)	2 (0.97)	3 (3.16)	2 (1.00)
Residence, n (%)				
Rural	245(48.80)	91 (44.17)	62 (65.26)	92 (45.77)
Urban	257(51.20)	115 (55.83)	33 (34.74)	109 (54.23)
Smoking status, n (%)				
Non-smoker	278(55.38)	124 (60.19)	48 (50.53)	106 (52.74)
Former Smoker	54 (10.76)	14 (6.80)	15 (15.79)	25 (12.44)
Current Smoker	170(33.86)	68 (33.01)	32 (33.68)	70 (34.83)
15-7.4,14.1498ptAlcohol status, n (%)				
Never	301(59.96)	128 (62.14)	59 (62.11)	114 (56.72)
Occasional	132(26.29)	51 (24.76)	21 (22.11)	60 (29.85)
Frequent	69 (13.75)	27 (13.11)	15 (15.79)	27 (13.43)
SBP, mmHg (median, IQR)	140(126,157)	139.5 (125,158)	141 (125,156)	140 (128,156)
DBP, mmHg (median, IQR)	81 (73, 91)	80 (73,90)	82 (74,89)	81 (74,92)
Initial NIHSS score, (median, IQR)	2 (1, 3)	2 (1–3)	2 (1–3)	2 (1–3)
Admission mRS, (median, IQR)	1 (1, 2)	1 (1–3)	2 (1–3)	2 (1–2)
D-dimer, (ug/mL)	0.34 (0.22,0.68)	0.35 (0.24,0.68)	0.33 (0.23,0.73)	0.32 (0.20,0.65)
Baseline blood glucose, (mmol/L)	5.65 (4.98,7.17)	5.88 (4.98,7.33)	5.59 (5.02,6.59)	5.60 (4.98,7.25)
LDL-C, (mmol/L)	2.83(2.24,3.53)	2.91 (2.26,3.56)	2.83 (2.10,3.33)	2.79 (2.26,3.61)
HDL-C, (mmol/L)	1.04(0.86,1.24)	1.03 (0.88,1.25)	1.07 (0.86,1.21)	1.03 (0.85,1.24)
TG, (mmol/L)	1.33 (1.00,1.85)	1.31 (1.00,1.78)	1.29 (1.03,1.75)	1.38 (0.97,1.96)
TC, (mmol/L)	4.30 (3.62,5.03)	4.33 (3.62,5.03)	4.23 (3.44,4.94)	4.30 (3.64,5.08)
CREA, (umol/L)	74(61,87)	75(61,89)	72 (61,89)	74 (62,85)
Urea, (mmol/L)	5.20 (4.30,6.30)	5.40(4.40,6.50)	5.00 (4.25,5.90)	5.10 (4.30,6.30)
ALT, (IU/L)	19 (14, 27)	20 (15,26)	19 (14,25)	20 (14,29)
AST, (IU/L)	22 (18, 27)	22 (18,28)	22 (18,27)	22 (18,27)
IVT, n (%)	39 (7.77)	12 (5.83)	9 (9.47)	18 (8.96)
Antiplatelets use before stroke, n (%)				
No	411(81.87)	167 (81.07)	80 (84.21)	164 (81.59)
Yes	77 (15.34)	34 (16.50)	11 (11.58)	32 (15.92)
Unknown	14 (2.79)	5 (2.43)	4 (4.21)	5 (2.49)
Anticoagulants use before stroke, n (%)				
No	465(92.63)	188 (91.26)	89 (93.68)	188 (93.53)
Yes	25 (4.98)	15 (7.28)	3 (3.16)	7 (3.48)
Unknown	12 (2.39)	3 (1.46)	3 (3.16)	6 (2.99)
Statin use before stroke, n (%)				
No	394(78.49)	159 (77.18)	77 (81.05)	158 (78.61)
Yes	89 (17.73)	40 (19.42)	12 (12.63)	37 (18.41)
Unknown	19 (3.78)	7 (3.40)	6 (6.32)	6 (2.99)
Wake-up stroke, n (%)	38 (7.57)	8 (3.88)	14 (14.74)	16 (7.96)
Stroke of unknown onset time, n (%)	32 (6.37)	15 (7.28)	5 (5.26)	12 (5.97)
Hypertension, n (%)	354(70.52)	152 (73.79)	65 (68.42)	137 (68.16)
Diabetes, n (%)	197(39.24)	92 (44.66)	32 (33.68)	73 (36.32)
Atrial fibrillation, n (%)	51 (10.16)	26 (12.62)	7 (7.37)	18 (8.96)
Ischemic heart disease, n (%)	41 (8.17)	14 (6.80)	9 (9.47)	18 (8.96)
Chronic heart failure, n (%)	17 (3.39)	8 (3.88)	4 (4.21)	5 (2.49)
Peripheral artery disease, n (%)	11 (2.19)	3 (1.46)	4 (4.21)	4 (1.99)
Stroke location, n (%)				
Anterior	297(59.40)	124 (60.19)	54 (56.84)	119 (59.20)
Posterior	148(29.60)	57 (27.67)	29 (30.53)	62 (30.85)
Mixed	55 (11.00)	24 (11.65)	11 (11.58)	20 (9.95)
TOAST subtypes, n (%)				
LAA	162(32.40)	72 (34.95)	38 (40.00)	52 (25.87)
CE	48 (9.60)	26 (12.62)	6 (6.32)	16 (7.96)
SAO	214(42.80)	83 (40.29)	36 (37.89)	95 (47.26)
SOE	25 (5.00)	6 (2.91)	7 (7.37)	12 (5.97)
SUE	51 (10.20)	18 (8.74)	7 (7.37)	26 (12.94)
6-month mRS score n (%)				
mRS of 0	140 (27.89)	60 (29.13)	26 (27.37)	54 (26.87)
mRS of 1	209 (41.63)	76 (36.89)	38 (40.00)	95 (47.26)
mRS of 2	68 (13.55)	30 (14.56)	10 (10.53)	28 (13.93)
mRS of 3	51 (10.16)	24 (11.65)	12 (12.63)	15 (7.46)
mRS of 4	33 (6.57)	15 (7.28)	9 (9.47)	9 (4.48)
mRS of 5	1 (0.20)	1(0.49)	0 (0)	0 (0)
mRS of 6	0 (0)	0 (0)	0 (0)	0 (0)
mRS of 0-1	349 (69.52)	136 (66.02)	64 (67.37)	149 (74.13)
mRS of 0-2	417 (83.07)	166 (80.58)	74 (77.89)	177 (88.06)

No deaths occurred among the 502 participants included in the final analysis. For the 60 participants excluded due to loss to follow-up or withdrawal, vital status could not be fully ascertained; however, no deaths were documented in medical records or death registry for this group. IQR, Interquartile Range; NIHSS, National Institutes of Health Stroke Scale; TOAST, Trial of Org 10,172 in Acute Stroke Treatment; IVT, intravenous thrombolysis; LAA, large-artery atherosclerosis; SE, cardioembolism; SAO, small-artery occlusion; SOE, acute stroke of other determined etiology; SUE, stroke of undetermined etiology; SBP, systolic blood pressure; DBP, diastolic blood pressure; mRS, Modified Rankin Scale; TC, total cholesterol; TG, triglycerides; LDL-C, low density lipoprotein cholesterol; HDL-C, High density lipoprotein cholesterol; CREA, Creatinine; Urea, Urea Nitrogen; ALT, Alanine Aminotransferase; AST, Aspartate Aminotransferase.

## Association between PA trajectories and functional outcomes

The overall three-level test for the trajectory variable was not statistically significant (*P* = 0.086). In an exploratory pairwise comparison, compared with decreased PA, increased PA was associated with a higher odds of achieving 6-month functional independence (mRS 0–2). After adjustment for age, sex, diabetes, atrial fibrillation, and hypertension, the adjusted OR for increased PA was 2.03 (95% CI 1.05–3.90, *P* = 0.034) for mRS 0–2 (Table 2). The corresponding adjusted Risk Ratio (aRR) was 1.13, indicating a 13% relative increase in the probability of functional independence for the increased group compared to the decreased group. In the secondary analysis using mRS 0–1 as the outcome, the overall three-level test for the trajectory variable was not significant (*p* = 0.370), and the pairwise comparison between Increased and Decreased was also not significant (OR: 1.35; 95% CI: 0.78–2.32; *P* = 0.283; Supplementary Table S4).

**Table 2:** Association between physical activity trajectories and functional outcomes.

Physical activity trajectory	Model 1			Model 2			Overall three-level *p*-value
	OR	95% CI	*p*-value	Adjusted OR	95% CI	*p*-value	
**mRS 0-2 at 6 months**							**0.086**
Decreased		Ref			Ref		
Stable low	1.18	0.65, 2.14	0.590	1.29	0.70, 2.36	0.418	
Increased	2.09	1.10, 3.99	0.025	2.03	1.05, 3.90	0.034	

Model 1 no adjustment; Model 2 adjusted for age, sex, diabetes, atrial fibrillation, and hypertension. OR, Odds Ratio, CI, Confidence Interval, Ref, Reference.

## Subgroup analyses

Subgroup analyses stratified by age and sex showed that the association between physical activity trajectories and 6-month functional independence was generally consistent across subgroups. No significant interactions were detected by age or sex (Figure 5).

**Figure 5:**
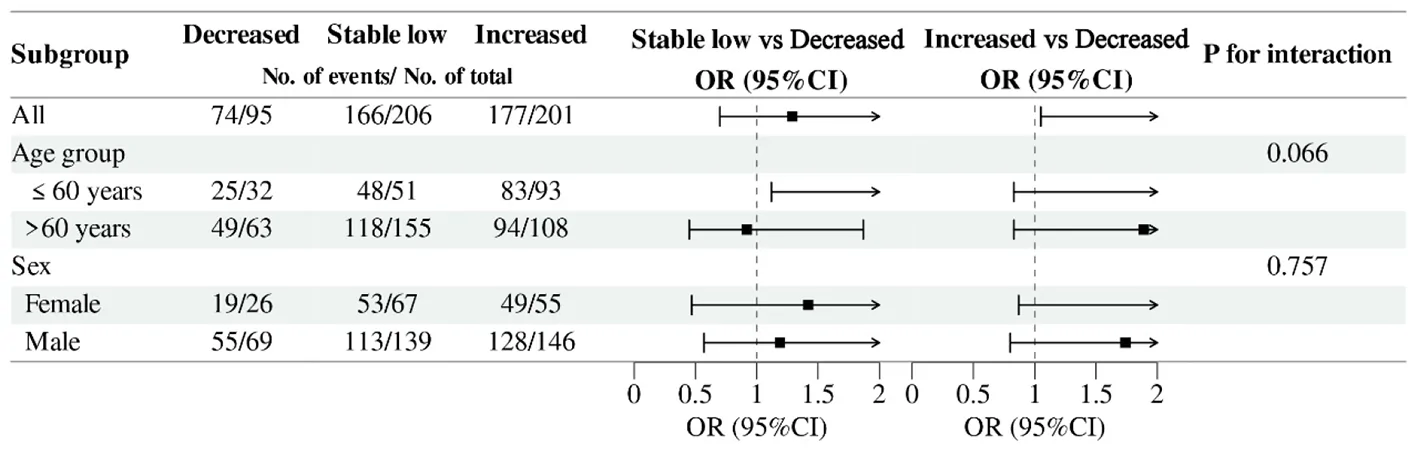
Subgroup analyses for the association between PA trajectories and 6-month functional independence (mRS 0–2). Estimates are presented as adjusted odds ratios with 95% confidence intervals. The reference group is the decreased trajectory. All estimates were adjusted for age, sex, hypertension, diabetes, and atrial fibrillation. *P* values for interaction were derived from Wald tests. Subgroup analyses by age (< 60 vs. ≥60 years) and sex (male vs. female) were exploratory and not pre-specified.

## Sensitivity analyses

In a sensitivity analysis additionally adjusting for admission mRS, NIHSS, thrombolysis, and wake-up stroke (Table 3), the results remained consistent with the primary findings (adjusted OR for increased vs. decreased: 2.50; 95% CI: 1.19–5.27; *P* = 0.016). The overall three-level test for the trajectory variable was not statistically significant in the primary model (*P* = 0.086) but reached nominal significance in the sensitivity analysis (*P* = 0.045). Pairwise comparisons should be interpreted as exploratory. E-value analysis for the primary comparison (adjusted OR = 2.03; 95% CI: 1.05–3.90) yielded an E-value of 1.51 for the point estimate and 1.11 for the lower confidence limit. These values suggest that the observed association could be sensitive to relatively weak unmeasured confounding; therefore, residual confounding cannot be entirely ruled out. E-values address only unmeasured exposure-outcome confounding and do not address reverse causation, selection bias, measurement error, or time-varying confounding (Supplementary Table S5). Ordinal logistic regression showed that higher SGPALS levels were associated with higher odds of functional independence in the crude model, with level 2 significantly associated with favorable outcomes compared with level 1 (crude OR: 1.83; 95% CI: 1.06–3.17; *P* = 0.030). After adjustment for age, sex, hypertension, diabetes, and atrial fibrillation, the association was attenuated and no longer statistically significant (adjusted OR: 1.66; 95% CI: 0.94–2.90; *P* = 0.078; Supplementary Table S6). In the posterior-probability-weighted sensitivity analysis, the direction of association was consistent with the primary analysis (Stable low vs. Decreased: OR = 1.26, 95% CI 0.75–2.13, *P* = 0.385; Increased vs. Decreased: OR = 1.67, 95% CI 1.01–2.78, *P* = 0.045; Supplementary Table S8). This analysis should be interpreted as a sensitivity analysis, not as a formal correction for classification error.

**Table 3:** Primary and sensitivity analyses of the association between physical activity trajectories and 6-month functional independence (mRS 0–2).

Physical activity trajectory	Model 2 (Primary)				Model 3 (Sensitivity)			
	OR	95% CI	*p*-value	Overall *p*-value	Adjusted OR	95% CI	*p*-value	Overall *p*-value
Decreased		Ref		0.086		Ref		0.045
Stable low	1.29	0.70, 2.36	0.418		1.50	0.74, 3.05	0.264	
Increased	2.03	1.05, 3.90	0.034		2.50	1.19, 5.27	0.016	

Model 2 adjusted for age, sex, diabetes, atrial fibrillation, and hypertension. Model 3 additionally adjusted for admission mRS, NIHSS, intravenous thrombolysis, and wake-up stroke. The overall three-level test for the trajectory variable was not statistically significant in Model 2 (*P* = 0.086) but reached nominal significance in Model 3 (*P* = 0.045). Pairwise comparisons should be interpreted as exploratory. Abbreviations: OR: Odds Ratio, CI: Confidence Interval, Ref: Reference.

## Discussion

In this study of patients with mild ischemic stroke, three exploratory physical activity trajectory classes from pre-stroke to 3 months post-stroke were identified. The overall three-level test was not significant in the primary model. In an exploratory pairwise comparison, the increased trajectory was associated with better functional independence compared with the decreased trajectory, whereas the stable low trajectory did not differ significantly from the decreased trajectory. The three-class trajectory solution should be considered exploratory, as it was not supported by an ordinal trajectory model.

Our analyses identified three PA trajectories after mild stroke, suggesting that post-stroke PA among patients with mild ischemic stroke does not follow a single uniform pattern but instead exhibits marked heterogeneity in trajectory. This observation is consistent with previous longitudinal evidence. In the Norwegian Cognitive Impairment After Stroke Study, four distinct long-term physical activity trajectory groups were identified, including a subgroup with persistently low and declining activity (10). Similarly, a longitudinal study of 523 patients with first-time ischemic stroke showed that although no overall change in PA was observed at the group level during 6 months of follow-up, substantial individual variation existed, with some patients increasing and others decreasing their activity levels (11). All aforementioned findings support the notion that PA after stroke may follow multiple behavioral courses rather than a uniform recovery pattern.

In our cohort, the overall trajectory test was not significant; however, in an exploratory pairwise comparison, increased PA was associated with a higher likelihood of achieving functional independence (mRS 0–2) at 6 months, whereas the stable low trajectory did not differ significantly from the decreased trajectory. This heterogeneity in PA trajectories may also help explain why patients experience different functional outcomes after mild ischemic stroke. Our findings are in line with previous studies indicating that higher levels of PA were associated with better functional outcomes after stroke (12, 30). Evidence from randomized controlled trials (31) showed that PA intensity during the acute stage of stroke rehabilitation can influence recovery, suggesting that greater exposure to PA may be associated with functional improvement through repeated practice, cardiovascular conditioning, and neuroplastic adaptation. In addition, the favorable outcomes observed in the increased PA group may partly reflect better adherence to rehabilitation-related PA. Findings from the Life After Stroke (LAST) study (30) showed that patients who adhered more closely to prescribed PA and exercise programs achieved better functional recovery, indicating that sustained behavioral engagement may be an important mechanism linking more favorable PA trajectories to improved outcomes.

We acknowledge that the observed association may be alternatively explained by recovery status enabling physical activity, rather than physical activity promoting recovery. Patients with better functional recovery by 3 months may simply be more capable of increasing their PA, whereas those with poorer recovery may not. Although our temporal design—with the exposure (T0–T2) preceding the outcome (T3)—partially addresses reverse causation, residual reverse causality cannot be entirely excluded, as the 3-month PA measurement may itself be influenced by the patient's functional status at that time, which we did not formally measure. Future studies with repeated intermediate functional assessments are needed to disentangle these bidirectional relationships.

Our study also adds to the existing literature by moving beyond single time-point or average measures of physical activity and characterizing its longitudinal change over time. Prior to our study, studies focusing on patients with mild ischemic stroke were rarely found. Although the mild stroke population is generally considered to have a favorable prognosis, our findings show that meaningful heterogeneity in activity recovery still exists and may have clinical relevance. These results highlight the potential value of characterizing early activity patterns to inform rehabilitation strategies. Notably, we did not observe a significant difference between the stable low and decreased trajectories, which differs somewhat from previous reports. This discrepancy may relate to differences in study population, activity measurement, follow-up duration, and analytic methods. In particular, our cohort included only patients with mild stroke, who generally had good outcomes, which may have limited between-group differences, and the 6-month follow-up may not have been long enough to capture the cumulative impact of declining activity.

Our study was highlighted by characterizing longitudinal PA trajectories rather than relying on single time-point measures among patients with mild ischemic stroke, a relatively understudied population. We acknowledge limitations. First, the generalizability of our findings is limited and causal inference cannot be established due to the single-center observational design. Since our cohort included only patients with first-ever mild ischemic stroke, these findings may not be directly generalizable to those with moderate or severe stroke. Second, we did not measure time-varying confounders such as functional status, rehabilitation exposure, cognition, depression, fatigue, or mobility limitations during follow-up; these factors may influence both PA levels and outcomes. Third, we excluded 60 participants who did not have complete pre-stroke, 1-month, and 3-month PA data or a 6-month mRS outcome. Based on medical record review and death registry linkage, no deaths were documented among these participants; however, detailed baseline characteristics and 6-month functional outcomes were not available for them due to loss to follow-up or withdrawal. The exclusion of these participants may introduce selection bias, and we were unable to perform a reliable Inverse Probability of Censoring Weighting (IPCW) analysis due to the limited number of censored events and incomplete covariate data for this subgroup. Fourth, retrospective assessment of pre-stroke PA may be subject to recall bias, and differential reporting according to recovery status cannot be excluded. Fifth, the entropy of the trajectory model was moderate (0.520), indicating classification uncertainty. A formal classification-error correction was not reliably obtainable. We therefore performed a posterior-probability-weighted logistic regression as a sensitivity analysis. This analysis showed a consistent direction of association but attenuated effect sizes and does not fully correct for latent class misclassification. In addition, an ordinal trajectory model did not support a stable three-class solution. Therefore, the three-class trajectory solution and all related associations should be considered exploratory and require external validation. We have reported Average Posterior Probability (AvePP) values to indicate classification quality, but residual uncertainty cannot be entirely excluded (Supplementary Table S3). Sixth, PA was assessed using a scale-based measure rather than objective devices.

## Conclusions

In conclusion, PA trajectories following a mild ischemic stroke are heterogeneous. The overall three-level test was not significant in the primary model. In an exploratory pairwise comparison, the increased trajectory was associated with better functional independence compared with the decreased trajectory. These findings should be considered exploratory and require external validation; causality cannot be inferred.

## Data Availability

The raw data supporting the conclusions of this article will be made available by the authors, without undue reservation.

## References

[1] Wu L Wang A Wang X Zhao X Wang C Liu L . Factors for short-term outcomes in patients with a minor stroke: results from China National Stroke Registry. BMC Neurol. (2015) 15:253. doi: 10.1186/s12883-015-0505-z 26645312 PMC4673844

[2] Lavallee PC Charles H Albers GW Caplan LR Donnan GA Ferro JM . Underlying causes of TIA and minor ischemic stroke and risk of major vascular events. JAMA Neurol. (2023) 80:1199–208. doi: 10.1001/jamaneurol.2023.3344 37782494 PMC10546292

[3] Cucchiara B Elm J Easton JD Coutts SB Willey JZ Biros MH . disability after minor stroke and transient ischemic attack in the POINT trial. Stroke. (2020) 51:792–9. doi: 10.1161/STROKEAHA.119.027465 32078486 PMC7313376

[4] Cowan LT Tome J Mallhi AK Tarasenko YN Palta P Evenson KR . Changes in physical activity and risk of ischemic stroke: the ARIC study. Int J Stroke. (2023) 18:173–9. doi: 10.1177/17474930221094221 35361010 PMC9887651

[5] Reinholdsson M Palstam A Sunnerhagen KS. Prestroke physical activity could influence acute stroke severity (part of PAPSIGOT). Neurology. (2018) 91:e1461–e7. doi: 10.1212/WNL.0000000000006354 30232251 PMC6202943

[6] Hung SH Ebaid D Kramer S Werden E Baxter H Campbell BC . Pre-stroke physical activity and admission stroke severity: a systematic review. Int J Stroke. (2021) 16:1009–18. doi: 10.1177/1747493021995271 33527883

[7] Gothe NP Bourbeau K. Associations between physical activity intensities and physical function in stroke survivors. Am J Phys Med Rehabil. (2020) 99:733–8. doi: 10.1097/PHM.0000000000001410 32167953

[8] Li F Geng X Huber C Stone C Ding Y. In search of a dose: the functional and molecular effects of exercise on post-stroke rehabilitation in rats. Front Cell Neurosci. (2020) 14:186. doi: 10.3389/fncel.2020.00186 32670026 PMC7330054

[9] Cefis M Chaney R Wirtz J Meloux A Quirie A Leger C . Molecular mechanisms underlying physical exercise-induced brain BDNF overproduction. Front Mol Neurosci. (2023) 16:1275924. doi: 10.3389/fnmol.2023.1275924 37868812 PMC10585026

[10] Luzum G Allore H Han L Saltvedt I Tan X Thingstad P . The longitudinal course of physical activity behavior poststroke and its variation across subgroups: the Norwegian cognitive impairment after stroke study. Phys Ther. (2025) 105:pzaf069. doi: 10.1093/ptj/pzaf069 40360170 PMC12309381

[11] Damsbo AG Blauenfeldt RA Andersen G Johnsen SP Mortensen JK. Trajectories of physical activity after ischaemic stroke: exploring prediction of change. Eur J Neurol. (2025) 32:e16545. doi: 10.1111/ene.16545 39564872 PMC11625951

[12] Buvarp D Viktorisson A Axelsson F Lehto E Lindgren L Lundstrom E . Physical activity trajectories and functional recovery after acute stroke among adults in Sweden. JAMA Netw Open. (2023) 6:e2310919. doi: 10.1001/jamanetworkopen.2023.10919 PMC10152305 37126346

[13] Fang S Zhang B Lei H Zhang Y Lin H Chen Q . No sex difference in the association of pre-stroke physical activity with functional independence after ischemic stroke. Sci Rep. (2025) 15:14495. doi: 10.1038/s41598-025-93914-5 40281015 PMC12032208

[14] Viktorisson A Palstam A Nyberg F Berg C Lissner L Sunnerhagen KS. Domain-specific physical activity and stroke in Sweden. JAMA Netw Open. (2024) 7:e2413453. doi: 10.1001/jamanetworkopen.2024.13453 38809556 PMC11137634

[15] Finch E Foster M Fleming J Cruwys T Williams I Shah D . Exploring changing needs following minor stroke. Health Soc Care Community. (2020) 28:347–56. doi: 10.1111/hsc.12866 31568627

[16] Lim A Ma H Ly J Singhal S Pan Y Wang Y . Comparison of dual antiplatelet therapies for minor, nondisabling, acute ischemic stroke: a Bayesian network meta-analysis. JAMA Netw Open. (2024) 7:e2411735. doi: 10.1001/jamanetworkopen.2024.11735 38753327 PMC11099682

[17] Grimby G Borjesson M Jonsdottir IH Schnohr P Thelle DS Saltin B. The “Saltin-Grimby Physical Activity Level Scale” and its application to health research. Scand J Med Sci Sports. (2015) 25 Suppl 4:119–25. doi: 10.1111/sms.12611 26589125

[18] Haggag H Hodgson C. Clinimetrics: Modified Rankin Scale (mRS). J Physiother. (2022) 68:281. doi: 10.1016/j.jphys.2022.05.017 35715375

[19] Banks JL Marotta CA. Outcomes validity and reliability of the modified Rankin scale: implications for stroke clinical trials: a literature review and synthesis. Stroke. (2007) 38:1091–6. doi: 10.1161/01.STR.0000258355.23810.c6 17272767

[20] Quimpo NJ Steiner PM. Drawing credible directed acyclic graphs for causal inference. Psychol Methods. (2026). doi: 10.1037/met0000831. [Epub ahead of print]. 41854686

[21] Adams HP Jr., Biller J. Classification of subtypes of ischemic stroke: history of the trial of org 10172 in acute stroke treatment classification. Stroke. (2015) 46:e114–7. doi: 10.1161/STROKEAHA.114.007773 25813192

[22] Chinese Chinese Society of Cardiology Chinese Chinese Medical Association Hypertension Committee of Cross-Straits Medicine Exchange Association Cardiovascular Disease Prevention Rehabilitation Rehabilitation Committee Chinese Association of Rehabilitation Medicine. Clinical practice guidelines for the management of hypertension in China. Zhonghua Xin Xue Guan Bing Za Zhi. (2024) 52:985–1032. 7. doi: 10.3760/cma.j.cn112148-20240709-00377 39289992

[23] American Diabetes Association Professional Practice C. 2. Diagnosis and classification of diabetes: standards of care in diabetes-2024. Diabetes Care. 2024; 47:S20–S42. doi: 10.2337/dc24-S002 PMC10725812 38078589

[24] Guo J Li Z Gu H Yang K Liu Y Lu J . Prevalence, risk factors and prognostic value of atrial fibrillation detected after stroke after haemorrhagic versus ischaemic stroke. Stroke Vasc Neurol. (2024) 9:652–9. doi: 10.1136/svn-2023-002974 PMC11791629 38365316

[25] Heidenreich PA Bozkurt B Aguilar D Allen LA Byun JJ Colvin MM . 2022 AHA/ACC/HFSA guideline for the management of heart failure: a report of the American College of Cardiology/American Heart Association Joint Committee on clinical practice guidelines. Circulation. (2022) 145:e895–e1032. doi: 10.1161/CIR.0000000000001073 35363499

[26] Kwon JY Sawatzky R Baumbusch J Lauck S Ratner PA. Growth mixture models: a case example of the longitudinal analysis of patient-reported outcomes data captured by a clinical registry. BMC Med Res Methodol. (2021) 21:79. doi: 10.1186/s12874-021-01276-z 33882863 PMC8058975

[27] van der Nest G Lima Passos V Candel M van Breukelen GJP. An overview of mixture modelling for latent evolutions in longitudinal data: modelling approaches, fit statistics and software. Adv Life Course Res. (2020) 43:100323. doi: 10.1016/j.alcr.2019.100323 36726256

[28] White IR Royston P Wood AM. Multiple imputation using chained equations: Issues and guidance for practice. Stat Med. (2011) 30:377–99. doi: 10.1002/sim.4067 21225900

[29] VanderWeele TJ Ding P. Sensitivity analysis in observational research: introducing the E-Value. Ann Intern Med. (2017) 167:268–74. doi: 10.7326/M16-2607 28693043

[30] Gunnes M Indredavik B Langhammer B Lydersen S Ihle-Hansen H Dahl AE . Associations between adherence to the physical activity and exercise program applied in the last study and functional recovery after stroke. Arch Phys Med Rehabil. (2019) 100:2251–9. doi: 10.1016/j.apmr.2019.04.023 31374191

[31] Amanzonwe ER Noukpo SI Adoukonou T Bonnechere B Feys P Hansen D . Exercise intensity matters in the rehabilitation of stroke in the acute stage: a randomized controlled trial. Neurorehabil Neural Repair. (2025) 39:892–905. doi: 10.1177/15459683251356969 40788139

