# Supplementary material for: Physical activity trajectories after mild ischemic stroke and their association with 6-month functional outcomes
Source: Front Neurol. 2026 Oct 22;17:1919846. doi: 10.3389/fneur.2026.1919846 (PMC13735618; doi:10.3389/fneur.2026.1919846)
Supplement: Supplementary file 1 [file Supplementary_file_1.docx]

**Supplemental materials:**

**Supplementary Table S1. Multiple imputation details for baseline covariates.**

| Item | Description |
| --- | --- |
| Variables with missing values | D-dimer, baseline blood glucose, LDL-C, HDL-C, TG, TC, CREA, Urea, ALT, AST |
| Missingness proportions | 2.0% – 18.9% across these variables |
| Variables with complete data | Age, sex, hypertension, diabetes, atrial fibrillation, admission mRS, NIHSS, smoking, alcohol, etc. |
| Number of imputations | 20 |
| Imputation model | Predictive mean matching for continuous variables, including all covariates and the outcome as auxiliary variables |
| Pooling of estimates | Rubin's rules |
| Scope of imputation | Baseline covariates only (within the 502 participants who completed all PA assessments) |

Footnote: No missing data were present for the outcome (mRS at 6 months) or the PA measurements. Multiple imputation of item-level missingness within the 502 analyzed participants does not correct for selection bias arising from the exclusion of the 60 participants without complete follow-up.

**Supplementary Table S2. Comparison of available baseline characteristics between included and excluded participants.**

| Characteristic | Included (n=502) \| | Excluded (n=60) | P value |
| --- | --- | --- | --- |
| Age, years, median (IQR) | 66 (57–72) | 67 (57–71) | 0.65 |
| Male, n (%) | 354 (70.5) | 38 (63.3) | 0.24 |
| Admission mRS, median (IQR) | 1 (1–2) | 1 (1–2) | 0.78 |
| 6-month mortality, n (%) \| | 0 (0) | 0 (0)* | _ |

*No deaths were documented among the 60 excluded participants based on medical record review and death registry linkage.

Footnote: Detailed baseline characteristics and 6-month functional outcomes were not available for the excluded participants due to loss to follow-up or withdrawal. Only age, sex, admission mRS, and mortality data could be retrieved.

**Supplementary Table S3: Fit indices of GMM on physical activity trajectories in patients with Mild ischemic stroke.**

| Model | BIC | AIC | SABIC | LogL | LMR p | Entropy | Class (%) | AvePP |
| --- | --- | --- | --- | --- | --- | --- | --- | --- |
| 1 | 3653.91 | 3641.26 | 3644.39 | -1817.63 | — | — | 100.0 | — |
| 2 | 3572.05 | 3546.74 | 3553.01 | -1767.37 | <0.001 | 0.715 | 79.3%/20.7% | 0.95/0.78 |
| 3 | 3558.35 | 3520.38 | 3529.78 | -1751.19 | <0.001 | 0.520 | 41.0%/40.0%/18.9% | 0.77/0.73/0.84 |
| 4 | 3577.01 | 3526.38 | 3538.92 | -1751.19 | 1.000 | 0.431 | 50.0%/31.1%/18.9%/0% | — |
| 5 | 3589.48 | 3526.20 | 3541.87 | -1748.10 | 0.118 | 0.483 | 0%/24.3%/55.2%/15.3%/5.2% | — |

Footnote: The 4-class and 5-class solutions were considered not admissible and were rejected due to the presence of empty classes (0%) and non-significant LMR tests. Abbreviations: BIC, Bayesian Information Criterion; SABIC, sample-size adjusted Bayesian information criterion; AIC, Akaike information criterion; LogL, Log-Likelihood; LMR p, Lo-Mendell-Rubin Test p-value; AvePP, Average Posterior Probability; GMM, growth mixture modeling.

**Supplementary Table S4. Association between physical activity trajectories and 6‑month excellent outcome (mRS 0–1).**

|  | **Model 1** | | |  | **Model 2** | | | **Overall three-level p-value** |
| --- | --- | --- | --- | --- | --- | --- | --- | --- |
|  | **OR** | **95% CI** | **p-value** |  | **Adjusted OR** | **95% CI** | **p-value** |  |
| **mRS 0-1 at 6 months** |  |  |  |  |  |  |  | 0.370 |
| Decreased |  | Ref |  |  |  | Ref |  |  |
| Stable low | 0.94 | 0.56,1.58 | 0.818 |  | 1.01 | 0.59, 1.71 | 0.981 |  |
| Increased | 1.39 | 0.81, 2.36 | 0.228 |  | 1.35 | 0.78, 2.32 | 0.283 |  |

Footnote: Model 1 no adjustment; Model 2 adjusted for age, sex, diabetes, atrial fibrillation, and hypertension. Abbreviations: OR: Odds Ratio, CI: Confidence Interval, Ref: Reference.

**Supplementary Table S5. E-value analysis for the association between physical activity trajectories and 6-month functional independence (mRS 0–2).**

| Comparison | Adjusted OR (95% CI) | Approximate RR* | E-value (point estimate) | E-value (lower CI) |
| --- | --- | --- | --- | --- |
| Increased vs Decreased | 2.03 (1.05–3.90) | 1.13 | 1.51 | 1.11 |

Footnote:*The approximate RR was calculated using the formula: RR ≈ OR / [(1−P0) + (P0 × OR)], where P0 is the outcome prevalence in the reference group (74/95 = 77.9%). E-values were calculated based on the approximate RR scale as recommended for common outcomes. Abbreviations: OR, odds ratio; CI, confidence interval; RR, risk ratio.

**Supplementary Table S6. Sensitivity analysis of the association between SGPALS category at 3 months post-stroke and 6‑month functional independence (mRS 0–2).**

|  | **Model 1** | | |  | **Model 2** | | |
| --- | --- | --- | --- | --- | --- | --- | --- |
|  | **OR** | **95% CI** | **p-value** |  | **Adjusted OR** | **95% CI** | **p-value** |
| SGPALS category (T2) |  |  |  |  |  |  |  |
| Level 1 |  | Ref |  |  |  | Ref |  |
| Level 2 | 1.83 | 1.06, 3.17 | 0.030 |  | 1.66 | 0.94, 2.90 | 0.078 |
| Level 3 | 1.77 | 0.90, 3.49 | 0.099 |  | 1.36 | 0.66, 2.80 | 0.403 |
| Level 4 | 0.68 | 0.20, 2.39 | 0.553 |  | 0.45 | 0.12, 1.70 | 0.241 |

Footnote: SGPALS level 4 had a very small sample size (n=13, 2.6% of the cohort), resulting in wide confidence intervals and imprecise estimates. Therefore, the non-significant OR for level 4 should be interpreted with caution and does not negate the overall positive trend observed across SGPALS levels 2 and 3. Model 1 no adjustment; Model 2 adjusted for age, sex, diabetes, atrial fibrillation, and hypertension. This analysis addresses whether treating SGPALS as ordinal versus continuous changes the association between SGPALS category and functional outcome. It does not test the stability of the latent trajectory solution. Abbreviations: OR: Odds Ratio, CI: Confidence Interval, Ref: Reference.

**Supplementary Table S7. DAGitty code for the directed acyclic graph (DAG).**

The DAGitty code (Supplementary Code S1) is as follows:

dag {

Trajectory [exposure, pos="4.000,2.000"]

mRS_6month [outcome, pos="6.000,2.000"]

Age [pos="-1.000,4.000"]

Sex [pos="-1.000,2.800"]

Hypertension [pos="-1.000,1.600"]

Diabetes [pos="-1.000,0.400"]

AtrialFibrillation [pos="-1.000,-0.800"]

PreStroke_PA [pos="1.000,4.000"]

Admission_mRS [pos="1.000,-2.000"]

NIHSS [pos="1.000,2.000"]

Thrombolysis [pos="1.000,0.500"]

WakeUpStroke [pos="1.000,-1.000"]

PA_1month [pos="3.000,4.000"]

PA_3month [pos="3.000,2.000"]

Censoring [pos="6.000,-1.000"]

Age -> Trajectory

Sex -> Trajectory

Hypertension -> Trajectory

Diabetes -> Trajectory

AtrialFibrillation -> Trajectory

Age -> mRS_6month

Sex -> mRS_6month

Hypertension -> mRS_6month

Diabetes -> mRS_6month

AtrialFibrillation -> mRS_6month

PreStroke_PA -> PA_1month

PreStroke_PA -> PA_3month

PreStroke_PA -> Trajectory

PreStroke_PA -> NIHSS

PreStroke_PA -> Thrombolysis

PreStroke_PA -> WakeUpStroke

Admission_mRS -> PA_1month

Admission_mRS -> PA_3month

Admission_mRS -> mRS_6month

NIHSS -> PA_1month

NIHSS -> PA_3month

NIHSS -> mRS_6month

Thrombolysis -> PA_1month

Thrombolysis -> PA_3month

Thrombolysis -> mRS_6month

WakeUpStroke -> PA_1month

WakeUpStroke -> PA_3month

WakeUpStroke -> mRS_6month

PA_1month -> Trajectory

PA_3month -> Trajectory

Trajectory -> mRS_6month

Censoring -> mRS_6month

}

The DAG structure was first generated using the R package dagitty (version 0.3-4). The R code defined all variables and causal relationships (directed arrows) based on temporal ordering. The resulting DAG structure was then visualized and refined in PowerPoint to ensure clarity and readability for clinical readers, with variables arranged according to their temporal position (pre-stroke → acute-phase → post-stroke PA → exposure → outcome).

For full reproducibility, we have provided the complete R/DAGitty code in the Supplementary Methods. The code can be directly pasted into the DAGitty web interface (http://www.dagitty.net) or run in R to reproduce the exact DAG structure. The primary model was adjusted for the following prespecified baseline covariates: age, sex, hypertension, diabetes, and atrial fibrillation. This analysis is described as a covariate-adjusted association, not as an estimate of a causal effect. We do not claim that these covariates constitute a minimal sufficient adjustment set. Post-stroke variables (admission mRS, NIHSS, thrombolysis, and wake-up stroke) were not included in the primary model; they were included together in a sensitivity analysis.

**Supplementary Table S8. Posterior-probability-weighted sensitivity analysis for the association between physical activity trajectories and 6-month functional independence (mRS 0–2).**

| Comparison | Adjusted OR (95% CI) | P value |
| --- | --- | --- |
| Increased vs Decreased | 1.67 (1.01–2.78) | 0.045 |
| Stable Low vs Decreased | 1.26 (0.75–2.13) | 0.385 |

Footnote: Model adjusted for age, sex, hypertension, diabetes, and atrial fibrillation. Each participant contributed three records corresponding to the three latent trajectory classes. The weight for each record was the posterior probability of class membership; weights summed to 1 per participant. Standard errors were cluster-robust by participant ID. Model adjusted for age, sex, hypertension, diabetes, and atrial fibrillation. This analysis is a sensitivity analysis incorporating posterior probabilities and is not a formal correction for latent class misclassification.
